# Inequalities in Resources for Preschool-Age Children by Parental Education: Evidence from Six Advanced Industrialized Countries

**DOI:** 10.1007/s10680-023-09685-0

**Published:** 2023-12-08

**Authors:** Jane Waldfogel, Sarah Jiyoon Kwon, Yi Wang, Liz Washbrook, Valentina Perinetti Casoni, Melanie Olczyk, Thorsten Schneider, Lidia Panico, Anne Solaz, Sabine Weinert, Anna Volodina, Sanneke de la Rie, Renske Keizer, Kayo Nozaki, Jun Yamashita, Yuriko Kameyama, Hideo Akabayashi

**Affiliations:** 1 Columbia University, 1255 Amsterdam Avenue, New York, 10027-5927 NY US; 2 University of Chicago, Chicago, IL US; 3 University of Bristol, Bristol, UK; 4 Martin Luther University Halle-Wittenberg, Halle, Saxony-Anhalt Germany; 5 Leipzig University, Leipzig, Saxony Germany; 6 Centre de Recherche Sur Les Inégalités Sociales (CRIS), CNRS, Sciences Po, Paris, France; 7 Institut National d’études Démographiques (INED), 93300 Aubervilliers, France; 8 University of Bamberg, Bamberg, Bavaria Germany; 9 Institute for Educational Quality Improvement at the Humboldt-Universität Zu Berlin, Berlin, Germany; 10 Erasmus University Rotterdam, Rotterdam, Netherlands; 11 Osaka University of Economics, Osaka, Japan; 12 Japan Women’s University, Tokyo, Japan; 13 Keio University, Tokyo, Japan; 14 Present Address: Hunter College, City University of New York, NY, USA

**Keywords:** Inequalities, Children, Family income, Center-based child care, Parental education, Comparative research

## Abstract

This paper provides new evidence on inequalities in resources for children age 3–4 by parental education using harmonized data from six advanced industrialized countries—United States, United Kingdom, France, Germany, Netherlands, and Japan—that represent different social welfare regime types. We analyze inequalities in two types of resources for young children—family income, and center-based child care—applying two alternative measures of parental education—highest parental education, and maternal education. We hypothesize that inequalities in resources by parental education will be less pronounced in countries where social policies are designed to be more equalizing. The results provide partial support for this hypothesis: the influence of parental education on resources for children does vary by the social policy context, although not in all cases. We also find that the measurement of parental education matters: income disparities are smaller under a maternal-only definition whereas child care disparities are larger. Moreover, the degree of divergence between the two sets of estimates differs across countries. We provide some of the first systematic evidence about how resources for young children vary depending on parents’ education and the extent to which such inequalities are buffered by social policies. We find that while early inequalities are a fact of life in all six countries, the extent of those inequalities varies considerably. Moreover, the results suggest that social policy plays a role in moderating the influence of parental education on resources for children.

## Introduction

Parental education is one of the strongest predictors of children’s life chances. Children of more educated parents are in better health and have higher levels of cognitive and behavioral skills during childhood, and higher levels of educational achievement and labor market success in adulthood (OECD, 2019).

Disparities in children’s health and development by parental education emerge early. Already at school entry, children of less-educated parents lag behind those of more educated parents (see, e.g., Bradbury et al., 2015). We know that parents with higher educational attainment are able to provide more resources and learning opportunities for their children than parents with lower educational attainment (Bassok et al., 2016; Byford et al., 2012). Yet, we still have much to learn about inequalities in resources in early childhood that may contribute to these disparities. This is an important gap in the literature as early childhood is a crucial period for child development and the establishment of unequal trajectories (Cunha et al., 2006).

In particular, we have much to learn about the extent to which inequalities in resources in early childhood by parental education are universal or vary by country context. While it has been argued that equalizing social policies can help break the link between parent status and resources for children (Esping-Andersen, 2004), to date there has been relatively little evidence on this. Comparative research can play a key role in advancing our understanding of the sources of and remedies for inequalities in child development, but also poses challenges, in particular the need for harmonized data and cross-country research teams (Lansford, 2016; Lansford et al., 2016; Waldfogel, 2013). Ideally, comparative research would utilize data on children’s outcomes (cognitive and socioemotional) measured prior to school entry, but the challenges of harmonization here are particularly acute^1^. In the absence of reliable comparative data on child outcomes prior to school entry, we can get closer to understanding the moderating role of country context for early inequalities by studying the distribution of key *inputs* that have been shown to be consequential for children’s development in the preschool period. This paper therefore provides new evidence on inequalities in resources among children age 3–4 using data from six advanced industrialized countries—United States, United Kingdom, France, Germany, Netherlands, and Japan—harmonized and analyzed by national researchers who are participants in the development of inequalities in child educational achievement: a six-country study (*DICE*) project.

The paper begins by reviewing the relevant literature. We next introduce the six countries and set out our main hypothesis. We then describe the data and methods, including the two measures of parental education and the two key types of early childhood resources—family income and center-based child care. We then present results for inequalities in these resources by parental education for children aged 3–4 in each of the six countries, before drawing conclusions.

## Literature Review

Many aspects of parents and families—aspects such as education, income, wealth, and occupation—are associated with social position for themselves and advantage or disadvantage for their children. Social scientists often summarize these under the rubric of socioeconomic status (SES; although this term is often used without being explicit about what is actually being measured, particularly in relation to children). Here, we focus on parental education as a measure of relative advantage/disadvantage for children because of its strong links with child development. While other aspects of SES are associated with child development, parental education has the strongest and largest effects on early cognitive outcomes (Hoff et al., 2012). It is also a good proxy for other important sources of advantage, in particular social and cultural as well as economic capital (Bradbury et al., 2015).

We are interested in the extent to which parental education is associated with more advantageous resources for children, starting in the early years. Our conceptual framework links children’s development, their home and out of home environments, and the wider social, institutional, and cultural influences on inequality emphasized in the sociology and policy literatures (Duncan & Murnane, 2011; Kalil, 2014; McLanahan, 2004; Putnam, 2015). Drawing on lifecycle models, human development is seen as a dynamic process in which a child’s skills and abilities depend on the level of skills and abilities the child has already acquired at an earlier stage and inputs the child experiences in the period in between; the early years are particularly crucial in this model because skills in early childhood lay the foundation for later learning (Cunha et al., 2006). We also draw on bioecological models of child development (Bronfenbrenner & Morris, 2006), which emphasize that distal factors at the family, community and societal levels shape the proximal environments experienced by children, which in turn affect their growth and development; such models particularly emphasize the influence of parents in the early years because of the predominant role they play in the inputs children receive.

We focus on early childhood because of this conceptual framework and because of evidence that inequalities in health and development between children from more and less advantaged backgrounds are already present before school entry (e.g., Duncan & Brooks-Gunn, 1997). Because of data limitations, we know relatively little from previous research about inequalities in family resources and experiences in early childhood, and how they vary across countries. Our study is designed to address that gap in knowledge. To that end, we provide new evidence on inequalities by parental education in two key types of resources for young children: (1) family income; and (2) center-based child care. As detailed below, we select these resources and experiences because they are likely to be correlated with parental education and to be consequential for children’s development. These resources are also relevant to policymakers as they can be influenced by policy measures such as universal child care, or taxes and transfers. In addition, as a practical matter, we focus on developmentally relevant items that are available and able to be harmonized across the six countries.

*Family income.* Parents with more education typically have higher incomes, because they earn higher returns in the labor market (see, e.g., Card, 1999) and because they are more likely to be partnered with more highly educated and higher earning spouses/partners (Cherlin, 2010; Komter et al., 2012). It is also well-established that higher family income is associated with better child development, with income in early childhood being particularly consequential, although much of the evidence derives from the USA (see, e.g., Cooper & Stewart, 2013; Duncan & Brooks-Gunn, 1997). Empirical evidence has shown that family income can impact child development through a range of mechanisms, which Duncan et al. (2014) summarize in terms of three complementary theoretical frameworks. The *family and environmental stress perspective* focuses on the effects of economic pressure and poor living conditions on levels of parental psychological distress, which are in turn linked to less nurturing parenting practices and adverse neurobiological consequences. The *resource and investment perspective* focuses on the role of income in facilitating investments in children in the form of enrichment experiences, quality of housing, nutrition, and so on. It also emphasizes that low income can be linked to the time parents have to spend with children, due to longer and less flexible work schedules. The *cultural perspective* emphasizes the effects of economic marginalization on parents’ values and beliefs that may then be transmitted to their children. Given this rich theoretical and empirical evidence base, there are good reasons to believe that the strength of the link between parental education and income across countries will have implications for education-related disparities in child development.

*Center-based child care.* Parents with more education are more likely to use non-parental child care for their young children and in particular are more likely to use center-based child care such as preschool or nursery school (Dearing et al., 2009). In part, this reflects such parents’ higher incomes and greater ability to pay for care, but may also reflect differences in preferences. Use of child care is also influenced by parental employment, especially employment of the primary caregiver (usually the mother). High-quality center-based child care, such as preschool or nursery school, has been shown to be positively associated with child development, across developed countries (Becker, 2011; Berger et al., 2021; Brooks-Gunn et al., 2002; Côté et al., 2013).

There is a disciplinary divide in the way parental education is conceptualized in relation to children’s attainment. The “dominance” approach is widely used in the status attainment literature from sociology, whereby parental education is defined as the highest educational qualification held by either of the child’s parents (Thaning & Hällsten, 2020; this literature also uses an “average” approach, whereby parental education is characterized as the average attainment across the two parents; we do not consider that approach here because our measures of parental education are categorical and not numeric years of education). In contrast, work from developmental psychology tends to focus on the education of the mother (Harding et al., 2015), emphasizing the assumed role of the mother as the primary caregiver and “central socializing influence” (Harding et al., 2015, p.73). Previous work on disparities in resources available to children by parental education has generally adopted either the dominance approach (e.g., Bradbury et al., 2015; McLanahan, 2004), or the maternal education approach (e.g., Crosnoe et al., 2021), without explicit consideration of this choice. Some studies have focused explicitly on the implications of the way maternal and paternal SES indicators, including education, are entered into models predicting offspring educational achievement or attainment (Erola et al., 2016; Korupp et al., 2002; Marks, 2008; Thaning & Hällsten, 2020). Typically, these studies find that multivariable specifications perform the best, such as the Modified Dominance model recommended by Korupp et al. (2002), in which an indicator of the education level of the lower-educated parent is added to supplement the indicator for the highest educated parent. There are two major problems with specifications that use separate indicators for each parent, rather than a single household-level measure of education. The first is that it requires different handling of one- and two-parent households as, by definition, single-parent households do not have an education level for the second partner; such specifications inherently pick up effects of family structure as well as education. The second problem, as recognized by Marks (2008), in part follows from the first, which is that “it makes cross-sample comparisons extremely difficult, so interesting questions on changes over-time or cross-national differences cannot be easily addressed” (p. 294). Given our focus on cross-national comparisons in this study, we restrict our attention to two alternative household-level measures that can be applied equally to one- and two-parent families and that generate single number summary measures of inequalities that can be easily compared. That is, we take the highest educational qualification held by a parent co-resident with the child as our primary measure but assess the sensitivity of our results by repeating our analyses using the mother’s highest educational qualification.

It cannot be predicted a priori whether gaps will be larger under one definition than the other. In addition, it may be that the effects of maternal and paternal education on family resources are asymmetric, for example if paternal resources are relatively more salient for the generation of family income in the labor market and maternal resources for decisions about care arrangements for the child. Some evidence for the disproportionate importance of mothers’ education in the early years is provided by Erola et al. (2016), who show that, in a Finnish sample, the education of the mother has a stronger independent influence on offspring occupational status than that of the father in the early childhood period, whereas by early adulthood this is reversed. Most importantly for our purposes, the degree of association between the two household-level indicators and measures of family resources may differ across countries. Marks (2008) documents considerable cross-national variation in the relative predictive power of maternal and paternal education for test scores at 15 with, for example, maternal education having the relatively stronger effect in Germany but paternal education the stronger effect in the US. Further, the extent to which classification of households diverges under to the two measures will depend on the relative attainment levels of males and females in a country, the degree of assortative mating, and the rate of single parenthood. Given the different practices used in research on early childhood conditions to date, and the lack of systematic evidence on the sensitivity of cross-national comparisons in this period to the way in which parental education is defined, it is instructive to see whether a definition that excludes fathers results in substantively different conclusions about the patterning of resource disparities across countries.

We recognize that parental education is correlated with other demographic characteristics—parent age, marriage/partnership, immigrant background—that matter for child development and that may also affect the resources for children. The extent of these correlations may differ across countries, and differences in demographic composition could then partly account for cross-national differences in resource disparities. We therefore explore the extent to which resource disparities by education become more similar across countries after adjustment for these factors in a descriptive analysis.

Parent education is positively correlated with *parent age* and *marriage/partnership*, both of which have been linked to higher levels of investment in children and improved developmental outcomes (Hastings & Schneider, 2019). These associations have been reported across an array of countries, although patterns and magnitudes of effects differ across contexts (Raymo et al., 2015; Rendall et al., 2009). Associations between parental education and *immigrant background* are more complex. Both the direction and strength of the association between parental education and immigrant background vary considerably, depending on the country of origin of immigrants and their selection in terms of education within their country of origin (OECD, 2019). Links between parent immigration background and child development are also varied and complex: immigrants are not homogeneous, and immigrant background may confer both advantages and disadvantages for children depending on the context and the outcome considered (Crosnoe & Fuligni, 2012).

Another factor that potentially links parental education to resources for preschool-aged children is maternal employment. The incentive for a mother to work is positively related to her education level (via higher earnings) and negatively related to her partner’s education level (via an income effect) and to the cost of child care (via a price effect). More highly educated mothers typically work more (Steiber et al., 2016) and working mothers typically make greater use of non-parental child care, although whether this care is formal or informal differs by group and context (Cébrian et al., 2019). Incentives to work are also influenced by national contextual factors, such as the prevalence of family-friendly working practices, social norms and the gender wage differential (Gornick et al, 1998). It is therefore of interest to explore whether cross-national differences in resource disparities for children can be accounted for, in a descriptive sense, by differential rates of maternal employment between low- and high-educated groups.

We add to the small comparative literature on how family advantage relates to inequalities in the resources available to young children across countries^2^. Our study draws directly on Bradbury et al. (2015), which provided evidence that the links between parent education and resources for children at age 5–6 varied considerably across the four Anglo-American countries they examined, with the largest inequalities in family resources (such as income) generally found in the USA and UK and smaller inequalities seen in Canada and Australia, a pattern that was also reflected in inequalities in child development at that age. We also build on Waldfogel and Washbrook (2011a, 2011b), who examined gaps by family income among children aged 4–5 in the USA and UK; Bradbury et al. (2012), who examined gaps by parental education and income among children aged 4–5 in the US, UK, Canada, and Australia; and Bradbury et al. (2019), who examined gaps by family income among children aged 4–5 in the US, UK, Canada, and Australia.

Our study also draws on Crosnoe et al. (2021), who examined gaps in enrollment in center-based child care by maternal education in four Anglo-American countries (US, UK, Australia, and Ireland). In line with the concept of contingent protection (Fomby et al., 2011) which posits that inequalities can be moderated by social policies, the authors found that inequalities in enrollment between children of college-educated mothers vs. those without college were moderated by social policy context (as measured by the share of the country’s gross domestic product (GDP) spent on programs for families and children).

Our study differs from Bradbury et al. (2015) and Crosnoe et al. (2021) by including a larger number of countries from a wider variety of welfare state regime types, which differ considerably in their policy contexts for young children and their families. In common with Crosnoe et al. (2021) but in contrast with Bradbury et al. (2015), we examine data for preschool-age children, a group that has been little studied and for whom the influence of parental education may be stronger than for their older peers who spend more time out of the home in school or other community settings. We build on and extend the work by Crosnoe et al. (2021) by applying two distinct and more detailed measures of parental education and by examining gaps in family income as well as child care enrollment.

## The Six Countries

Our main hypothesis is that, in addition to augmenting resources for children overall, social policies can also close gaps between children from more versus less advantaged families, by providing income supports to less advantaged families and/or by providing services such as child care directly or reducing the cost of such services. In this sense, policies can be equalizing—“cutting the Gordian knot of inheritance” as Esping-Anderson (2004) put it (see also Fomby et al., 2011; Crosnoe et al., 2021). To test this hypothesis, we analyze data from six wealthy countries, which show large differences in their social policies and living conditions of families and resources for children in early childhood.

According to Esping-Andersen (1990), countries can be assigned to different welfare regimes, which vary with regard to the responsibilities of the state, market, and family in providing welfare and buffering against social risks. Welfare regimes differ for example in the way the unemployed are supported, mothers are integrated in the labor market, and benefits and services are provided to all persons (universal), targeted to the neediest only, or depend on (occupational) status. Welfare regimes also differ in their approach to child care (Blau & Kahn, 2013; Orloff, 2009) and the extent to which young families are targeted relative to other population groups such as the elderly. Crucially, welfare regimes affect inequality within countries, as they differ in the extent to which governments redistribute income. Variation in the details of welfare policies mean that equalizing effects for the population of families with young children may differ from those for other population groups, even within the same regime type (Gornick et al., 1998).

The USA and UK belong to the Anglo-American welfare regime. Government intervenes little, assuming that citizens can meet their needs in the market (Blossfeld, 2016, p. 59; Esping-Andersen & Myles, 2009, p. 645). Government supports are seen as residual and mainly go to those who are hard up, particularly in the USA (Katz, 2013). In the US, child care is mostly market based and fragmented. Parents can use tax credits, and there are some subsidies and special programs such as Head Start for those on low income. But universal provision is rare, except in the case of universal prekindergarten which now serves about one-fifth of 4 year olds (and a smaller share of 3 year olds; Friedman-Krauss et al., 2020). The UK historically resembled the USA in its approach to child care but in recent decades has adopted a more Continental European approach, instituting universal child care for three and 4 year olds (and some 2 year olds; Waldfogel, 2010).

France, Germany, and the Netherlands belong to the Continental European welfare regime. This regime is characterized by a comprehensive system of social insurance linked to occupation and status, with the family playing a central role for provision of welfare and the state subsidizing the family (Esping-Andersen & Myles, 2009, p. 647). There is considerable heterogeneity between countries in this welfare regime, particularly with regard to child care. France has long been the country with the most extensive universal provision, starting with highly subsidized creches as well as subsidized state-regulated caregivers who provide care in their home (“assistantes maternelles”) for infants and toddlers (Berger et al., 2021) and then universal and free ecoles maternelles from age three. In contrast, both Germany and the Netherlands adopted universal child care later than France, reflecting their greater reliance on the family for the care of children (Korpi et al., 2013), particularly in the first 3 years of life (Kosloski et al., 2016; Saraceno, 2011).

Japan, our sixth country, represents the East Asian regime type, which like countries in the Anglo-American group encourages provision of services by firms and the family. Social insurance and other benefits depend strongly on being employed in a large firm and having a high level of tenure (Blossfeld, 2016, referring to Esping-Andersen, 1990). Employment remains highly gendered, with a strong emphasis on women staying home to take care of children, particularly in early childhood (Raymo & Lim, 2011). Japan was a relative latecomer to universal child care.

How do our countries line up on key social policies that would be associated with more equal resources for children in early childhood? We focus on two metrics that have received considerable attention in the welfare state literature: income inequality and public expenditures on children. Overall income inequality can be thought of as composed of two components—inequality that occurs between education groups and within-education-group inequality. Our assumption is that countries with higher overall inequality will also tend to have higher between-group inequality because education-related earnings premiums themselves contribute to the degree of dispersion in incomes. It is possible, however, that education is a more important determinant of incomes in some countries than others, which would weaken the relationship between overall income inequality and education-related income gaps. The results of VanHeuvelen (2018), although defining between-group inequality in terms of a broader range of factors than just education, are supportive of both points. Between-group market earnings inequality is positively correlated with overall inequality, but its contribution differs across countries, playing a greater role in Continental European and Nordic welfare regimes than in the Anglo-American regime. Our hypothesis is that between-education-group income inequalities will be attenuated to differing degrees depending on a country’s level of social expenditure. However, cross-national differences in education-related income inequalities may also reflect market forces and labor market factors such as centralized wage bargaining. Some indication of the relative variation in the two sources of inequality across countries can be seen by comparing measures of income inequality calculated pre- and post-taxes and transfers.

Table 1 displays data on these metrics for the six countries, in each case drawing on information for the year when our sample children were age 3/4. (Table 1 also displays the OECD average in the latest available year for each metric, for comparison).

**Table 1:** Inequality and family-related public expenditures, by country/cohort

	Country (year of measurement)						
	US (2005)	UK (2004)	FR (2014)	GE (2015)	NL (2010)	JP (2012)	OECD (2017)
Income inequality pre-tax and transfers^a^	0.49	0.50	0.51	0.50	0.42	0.49	na
Income inequality post-tax and transfers^a^	0.38	0.33	0.29	0.29	0.28	0.33	0.32
Child poverty rate^b^	20.6	12.7	11.5	11.2	9.6	16.3	12.6
ECEC spending (% GDP)	0.33	0.76	1.31	0.60	0.83	0.38	0.70
Non-ECEC family spending^c^ (% GDP)	0.38	2.12	1.70	1.64	0.70	0.77	1.42

Data are presented for the year corresponding to the age 3–4 wave of data collection in each country cohort study (2012 is used for Japan rather than 2013 due to greater data availability). The most up to date statistics available for the OECD as a whole are presented for comparison. na indicates not available. *Sources* OECD Statistics, OECD Family Database

^a^Gini coefficients. Estimates for France and Germany use a slightly different income definition as a result of methodological changes in 2012. Pre-tax Gini for OECD as a whole not available

^b^The child poverty rate is set at 50% of median household income, post-tax and transfer

^c^Non-ECEC family spending is comprised of family allowances, maternity and parental leave, other cash benefits, home help/accommodation, and other in-kind benefits

*Income inequality.* One measure of a more equalizing social policy regime is how inequality in post-tax and -transfer income compares to inequality in market income (before taxes and transfers). We find that market income inequality measured by the Gini coefficient was very similar in the six countries (being slightly lower in the Netherlands only; Table 1), but inequality in post-tax and -transfer income varied considerably: it was highest in the countries from the Anglo-American welfare regime, the USA and UK (Gini of 0.38 and 0.33 respectively), and Japan (0.33), while the three countries from the Continental European welfare regime had lower inequalities (0.28–0.29) than the first three (and lower than the OECD average). This pattern is also seen in the proportion of children in poverty (defined as having less than 50% of median household income): 21% of children were in poverty in the US, 16% in Japan, 13% in the UK, versus 10–12% in the three Continental European countries. The similarity in market Gini coefficients suggests that inequality due to market forces differed very little across these six countries: variation in disposable income inequality was due almost entirely to variation in redistribution on the part of the state.

*Public expenditures on children*. Another important measure of a more equalizing social policy regime is the extent of public expenditures on children. Looking first at the share of GDP spent on early childhood education and care (ECEC), we find the highest share in France (1.3%) followed by the NL (0.8%) and UK (0.8%), with Germany (0.6%), Japan (0.4%), and the USA (0.3%) allocating less than the first three and less than the OECD average (0.7%). The percent of GDP spent on other family benefits (such as family allowances, maternity and parental leave, and other cash benefits) is by far lowest in the US, but highest in the UK.

Given this variation in social policies and our hypothesis about the potentially equalizing role of such policies, what might we expect in terms of how gaps in resources in early childhood differ across the six countries? Specifically, what patterns would we expect to see across countries for the two outcomes we consider—family income, and center-based child care?

With regard to income, the USA stands out with the highest level of post-tax and -transfer income inequality and child poverty and the lowest rate of public spending for children. Thus, we would expect gaps in income by parental education to be the largest there. The other Anglo-American regime country, the UK, has lower income inequality and child poverty and a higher rate of public spending for children; thus, we would expect gaps in income among families with preschool children to be smaller there than in the US. We would also expect smaller gaps in income in the three Continental European countries, all of which feature lower levels of income inequality and child poverty and higher rates of public spending (particularly in France) than the US. Finally, Japan’s policy regime looks more equalizing than the US, but less equalizing than the UK, on most indicators, so we would expect gaps in income to be smaller there than in the USA but larger than in the UK.

With regard to center-based child care, the most relevant policy metric is the share of GDP spent on ECEC (although other policy measures such as the share of GDP spent on other services or income supports might also matter). Here again the USA stands out—with the lowest share of GDP spent on ECEC—and we would expect gaps in center-based care to be the largest there. France in contrast stands out with the highest share of GDP allocated to ECEC, so we would expect gaps in center-based care to be the smallest there, while the Netherlands, UK, Germany, and Japan would be expected to occupy an intermediate position.

## Data and Methods

This paper draws on very rich data on young children and their families harmonized and analyzed by the development of inequalities in child educational achievement: a six-country study** (DICE)** project. Here we provide a brief outline of the data used from the six countries (for more details, see Table A1 in the online Appendix).

- United States (US): Early Childhood Longitudinal Study—Birth Cohort (ECLS-B; Snow et al., 2007); children born in 2001; early childhood waves at 10, 24 and **53** months (*N* = 8050; all ECLS-B sample sizes rounded to the nearest 50 in accordance with NCES regulations).
- United Kingdom (UK): Millennium Cohort Study (MCS; Connelly & Platt, 2014; University of London, 2021); children born in 2000; early childhood waves 10, **38** and 62 months (*N* = 15,552).
- France (FR): French Longitudinal Study of Children (ELFE; Charles et al., 2020); children born in 2011; early childhood waves at 0, 2, 13, 25, and **42** months (*N* = 11,071).
- Germany (GE): National Education Panel Study—Starting cohort 1 (NEPS-SC1; Blossfeld & Roßbach, 2019); children born in 2012; early childhood waves at 7, 14, 27, **39**, and 50 months (*N* = 2,474).
- Netherlands (NE): Generation-R (Gen-R; Jaddoe et al., 2012); children born in Rotterdam from 2002 to 2006; early childhood waves at 6, 12, 18, 24, **37** and 49 months (*N* = 4941).
- Japan (JP): Longitudinal Study of Newborns 2010 Birth Cohort (LSN2010; Household Statistics Office, 2017); children born in 2010; early childhood waves at 6, 18, 30, 42, and 54 months (*N* = 28,976).

Unless otherwise noted, we analyze data pertaining to children and their families at the wave closest to age 3. The children’s average age at that wave (bolded above) ranges from 3.1 years in the Netherlands to 4.4 years in the US. We drop the small number of cases in each country where the child is not co-resident with at least one biological or adopted parent at the target wave. Where there was more than one cohort member per family included in the dataset (due to a multiple birth), we randomly retain one cohort member per family for consistency across countries. Sample sizes given above are the achieved samples after these restrictions at the selected age 3–4 waves. The cohorts we analyze are roughly contemporaneous, with birth years ranging from 2000 to 2012.

With the exception of the Netherlands, where the sample is drawn from births in Rotterdam (a large, diverse city), all the samples are designed to be nationally representative. The ECLS-B, MCS, ELFE, and NEPS-SC1 provide longitudinal weights and survey design variables that can be used to adjust estimates for complex sampling and attrition since baseline. All estimates from the ECLS-B, MCS, and ELFE apply these recommended weights and survey design variables for the age 3–4 wave. Estimates from the NEPS-SC1 use weights constructed by the DICE study team; these augment the official longitudinal weights provided by NEPS with calibration against characteristics of the national population derived from 2016 microcensus data (the raking procedure used to adjust the official NEPS weights is described in the DICE Technical Appendix, DICE, 2021). Neither Gen-R nor the LSN provide official longitudinal weights so here again we use weights constructed by the DICE study team to adjust for non-random attrition (see the DICE, 2021). Weighting adjustments are made via the svy command in Stata.

Survey weights adjust for unit non-response, i.e., attrition, at the target wave but not for item non-response on independent variables within the target wave. Appendix Table A1 shows that a complete cases analysis would result in the loss of between 9 and 45 percent of our potential samples. We therefore used multiple imputation in STATA (Raghunathan et al., 2001) to account for item non-response, imputing 20 datasets to ensure appropriate power. We included our dependent variables in the imputation process, which helped to predict missing values on the independent variables. We then deleted cases with originally missing values on dependent variables (i.e., multiple imputation, then deletion; von Hippel, 2007). The results of the subsequent analyses with 20 imputed datasets were automatically combined in STATA in accordance with Rubin’s formulas, using mi estimate. Complete case results using listwise deletion are presented in the supplementary materials (Appendix B, Table B1–B4) for comparison.

### Parental Education

The harmonization of different systems of national educational qualifications is challenging. The International Standard Classification on Education (ISCED) is commonly used for this purpose but in the case of these six countries, ISCED levels tend to equate qualifications that have quite different implications for life chances and family resources in different countries. We therefore developed our own coding system to categorize parental education after extensive discussion between the national teams.

We define *high education* as a first/bachelor’s university degree or higher, requiring 3–4 years of full-time study at the tertiary level, in all countries. The definition of *low education* differs between countries with comprehensive systems (i.e., little or no tracking below age 16; the US, UK, France, and Japan) and those with early tracking and a high degree of academic/vocational specificity (Germany and the Netherlands). For the first group, low education is defined as no qualification beyond the expected standard, i.e., the target of the education system for all children in compulsory education. In the US, Japan and France this is a high school diploma^3^; in the UK this is attainment of at least a grade C qualification at the end of compulsory schooling (age 16). In the second group, low education is defined as no attainment beyond the intermediate/junior secondary track. The *medium education* group is all those who do not fall in either the high or low categories. In the US, for example, this category would include those with some education beyond high school but without a bachelor’s degree.

Our primary measure of parental education is based on the highest level of education attained by a parent who is co-resident with the child at the age 3–4 survey wave. We test the sensitivity of our results using a second measure, based on the highest level of education attained by the child’s mother.

To construct our primary measure, families are coded as high, medium, or low parental education based on the highest level of education of a parent (or step-parent) who is co-resident with the child at the age 3–4 survey wave. Thus where there is only one resident parent, the family is categorized based on her or his level of education; where there are two resident parents, the family is categorized based on the more highly educated of the two.

To construct our second measure—maternal education—families are coded as high, medium, or low maternal education based on the education of the primary co-resident caregiver to the child at the age 3–4 survey wave. In the overwhelming majority of cases this is the child’s biological mother so we use the terms mother/maternal for brevity and for consistency with previous literature. Note that the two measures will differ only when the partner of the mother is more educated than the mother among children living in two-adult families, which in turn depends on the degree of assortative mating and the gender differential in educational attainment levels. The educational level of lone mothers is identical regardless of the measure used.

As shown in Table 2, the distributions of highest parental education are very similar in the US, UK, and France with families falling into one of three roughly equal-sized categories. In contrast, the low education group is noticeably smaller in Germany. The Netherlands and Japan have a high share of children with a highly educated parent, a pattern consistent with other international statistics.

**Table 2:** Distributions of parental education at age 3–4

	Country (year of measurement)					
	US (2005)	UK (2004)	FR (2014)	GE (2015)	NL (2009–12)	JP (2013)
Observed sample N	7950	15,499	11,071	2,468	4,297	27,639
*Highest parental qualification*						
High (%)^a^	33.4	33.6	40.2	31.0	55.8	50.6
Medium (%)	33.3	28.5	29.5	48.9	28.4	30.3
Low (%)	33.3	37.9	30.3	20.1	15.8	19.0
*Highest maternal qualification*						
High (%)	26.0	23.1	31.2	21.7	48.0	27.0
Medium (%)	31.3	25.2	28.4	51.7	32.1	41.7
Low (%)	42.7	51.7	40.4	26.6	19.9	31.4
Cases re-classified under maternal definition (%)	13.7	19.0	14.5	17.1	9.7	29.7

^a^Weighted percentages

The distributions of maternal education are notably different. In the US, UK, and France, the modal category for mothers is low educated, while in Germany and Japan the modal category is medium educated and in the Netherlands it is high educated. Comparing the two distributions reveals that a considerable share of families who are coded as medium or higher when we take the spouse or partner’s education into account are coded as less educated according to the mother’s education alone. The stability of classifications varies greatly across countries, from just 9.7% of families re-classified under the maternal definition in the Netherlands to 29.7% in Japan, suggesting a higher degree of assortative mating in the former than the latter. These differences raise the possibility that cross-country comparisons of resource disparities may be patterned differently, depending on the definition of parental education used. Further details of the distribution of category switches are provided in Appendix Table A2. In the USA and UK, the most common source of re-classification relates to medium-educated partners paired with low-educated mothers, whereas in the other four countries it is high-educated partners paired to medium-educated mothers.

### Measures of Family Resources and Family Demographic Characteristics

We calculate gaps by parental education in two key family resources: *family income;* and *center-based child care.* We estimate both raw gaps and gaps controlling for *family demographic characteristics* (detailed below).

*Family income.* For each country, we construct a continuous measure of family income post-tax and -transfers. Surveys differ in the way income was collected (e.g., in bands, including or excluding certain taxes or transfers), and in most cases considerable processing was required to harmonize the data (see DICE, 2021, for details). Derived measures of family income were then converted to 2017 values using a national price index and converted to US dollars using the OECD PPP index for 2017; and equivalized for household size by dividing by the square root of the number of persons in the household times 0.5. This adjustment provides incomes calibrated to those for a family of four in 2017 US dollars. For ease of interpretation, we conduct analyses of the logged value of this income variable (but also provide descriptive information in levels).

*Center-based child care.* Our datasets provide detailed data on child care, including information about attendance at a center-based setting (any arrangement not located in a private home, including creches, daycare centers, playgroups, nurseries and preschools) for at least 2 time points (and up to 4 time points) between the age of 6 months and 3–4 years. In our main results, we focus on center-based child care at age 3–4 (but we also provide some descriptive results for attendance between 6 months and age 3–4).

Country means (or proportions) for each of these variables are displayed in Table 3.

**Table 3:** Whole sample descriptive statistics

	Country (year of measurement)					
	US (2005)	UK (2004)	FR (2014)	GE (2015)	NL (2009–12)	JP (2013)
*Equivalized disposable income*						
Thousands 2017$ M (SD)	56.2 (35.0)	49.9 (35.1)	48.6 (33.7)	53.8 (25.1)	52.3 (25.1)	47.2 (26.3)
2017$, logged M (SD)	10.76 (0.61)	10.55 (0.80)	10.66 (0.53)	10.79 (0.47)	10.74 (0.52)	10.63 (0.54)
Center-based care at 3–4 years (%)	54.3	31.4	98.4	80.1	91.3	55.2
*Family structure*						
Two biological/adoptive parents (%)^a^	71.8	79.8	91.1	82.2	80.5	96.1
Single parent (%)	22.1	17.6	8.1	15.0	17.5	3.9
Step-family (%)	6.1	2.6	0.8	2.8	2.0	~
Foreign-born parent (%)^b^	20.6	14.8	24.1	36.6	35.9	2.4
*Mother’s age at birth of child*						
< 20 (%)	10.9	7.9	1.5	1.6	2.9	0.7
20–24 (%)	24.9	16.7	13.5	10.7	14.9	9.2
25–29 (%)	26.4	27.7	32.8	25.5	25.7	29.1
30–34 (%)	23.7	30.5	32.4	35.7	37.8	37.1
35 + (%)	14.1	17.2	19.8	26.5	18.6	24.0
*Maternal employment at 3–4 years (%)*^c^						
Not in work (%)	42.4	51.3	36.3	46.1	24.6	52.5
Part-time (%)	17.6	36.7	10.2	39.5	55.1	31.8
Full-time (%)	40.0	12.1	53.4	14.4	20.3	15.7

Weighted statistics. Statistics are calculated on the observed sample for each variable, prior to multiple imputation. See Appendix Table A3 for information on samples sizes and missing data. ~ no observations

^a^Includes non-biological parents for Japan only

^b^Defined as a parent with a foreign nationality for Japan and France

^c^Employment measured at age 2 for the Netherlands (latest available data). Full-time employment defined as 32 h or more per week (40 h for Japan)

*Family demographic characteristics.* In our multivariate models, we control for demographic characteristics that are correlated with both parental education and resources for children.

*Parent age.* We distinguish five categories based on the mother’s age at the time of the child’s birth: under 20; 20–24; 25–29; 30–34; 35 or more.

*Family structure.* We distinguish three categories, according to whether the child co-resides with: two biological or adoptive parents; a single parent (of any sex); or one biological/adoptive parent and one step parent^4^.

*Immigrant background.* This is a binary indicator for whether the child resides with at least one parent who was born outside the host country^5^.

*Maternal employment. We distinguish 3 categories: full-time; part-time; or not employed. *Country means (or proportions) for each of these variables are displayed in Table 3.

We do not control for racial or ethnic background in our main estimates because the relevant categories differ considerably across countries. However, in supplementary analyses we investigated the sensitivity of our findings to the inclusion of additional country-specific controls, e.g., controls for child race/ethnicity in the USA and for residence in the former East or West Germany in Germany (see Appendix Tables A4 and A7).

## Analytic Approach

We calculate inequalities in income and center-based child care by parental education, presenting results for three sets of models: no controls; controlling for age, family structure, and immigrant background; and controlling for those three characteristics plus maternal employment. We estimate each model twice—once using highest parental education as the measure of family advantage, and once using maternal education. The unconditional inequalities from the first model capture the overall association between parental education and resources. The latter two models are used to explore the extent to which differing associations of education with demographic factors and maternal employment patterns can account for cross-national variation in the overall association. As countries differ markedly in their demographic composition and early maternal employment patterns, we expect that country differences by education will become less marked when these factors are controlled. Our estimates are descriptive and we do not intend a causal interpretation of the conditional models. The conditional inequalities allow us to compare cross-national differences in education-related disparities net of these two potentially important factors.

Our main focus is on differences between children of highly educated parents and those of low-educated parents in the mean level of, or the proportion with, a given resource or experience, which we refer to as high-low (H/L) gaps. These results are displayed in Table 4 (income) and Table 5 (center-based child care). We also present information in the Appendix on differences between children of highly educated parents and those of medium-educated parents (high-medium (H/M) gaps), and differences between children of low-educated parents and those of medium-educated parents (low-medium (L/M) gaps). The gaps in family income are simply the parental education coefficients from linear regressions where log income is the dependent variable. The gaps in center-based child care participation are derived from logistic regression models and calculated as the mean differences in marginal predicted probabilities of participation for a specified reference case.

**Table 4:** High-low parental education gaps in log household income at age 3–4

	US	UK	FR	GE	NL	JP	6-country Average
*By highest parental qualification*							
Controls:							
None	0.97^*^ (0.94, 1.01)	1.00^*^ (0.95, 1.05)	0.58^*^ (0.56, 0.60)	0.79 (0.70, 0.87)	0.83 (0.78, 0.89)	0.46^*^ (0.44, 0.48)	0.77 (0.65, 0.90)
Demographics	0.76^*^ (0.72, 0.80)	0.66 (0.62, 0.70)	0.46 (0.44, 0.48)	0.50 (0.41, 0.60)	0.57 (0.50, 0.63)	0.38^*^ (0.37, 0.40)	0.56 (0.42, 0.69)
Demographics + employment	0.74^*^ (0.70, 0.78)	0.59 (0.54, 0.64)	0.41 (0.39, 0.43)	0.45 (0.36, 0.54)	0.45 (0.38, 0.51)	0.39 (0.37, 0.40)	0.50 (0.38, 0.63)
*By highest maternal qualification*							
Controls:							
None	0.93^*^ (0.90, 0.97)	0.86^*^ (0.81, 0.91)	0.55^*^ (0.53, 0.57)	0.74 (0.67, 0.82)	0.79 (0.74, 0.84)	0.44^*^ (0.43, 0.46)	0.72 (0.61, 0.83)
Demographics	0.72^*^ (0.68, 0.76)	0.58 (0.54, 0.63)	0.45 (0.43, 0.47)	0.49 (0.42, 0.57)	0.54 (0.49, 0.59)	0.38^*^ (0.37, 0.40)	0.53 (0.42, 0.64)
Demographics + employment	0.70^*^ (0.66, 0.74)	0.51 (0.46, 0.55)	0.40 (0.38, 0.42)	0.43 (0.36, 0.50)	0.44 (0.39, 0.49)	0.37 (0.36, 0.39)	0.47 (0.37, 0.58)
*N*	7450	13,100	10,980	2397	4558	26,563	–

Estimated are coefficients from linear regression models with log household income as the dependent variable. 95% confidence intervals in parentheses

^*^indicates significant difference from the 6-country average (*p* < .05). Demographic controls are: family structure; foreign-born parent; maternal age at birth. Employment controls are for no work, part-time or full-time maternal employment. See Table 3 for details. Multiple imputation is used to account for missing data on covariates

**Table 5:** High-low parental education gaps in participation in center-based care at age 3–4

	US	UK	FR	GE	NL	JP	6-country Average
*By highest parental qualification*							
Controls:							
None	0.42^*^ (0.39, 0.45)	0.22 (0.19, 0.25)	0.02^*^ (0.01, 0.03)	0.22 (0.14, 0.31)	0.11 (0.07, 0.16)	-0.01^*^ (-0.03, 0.00)	0.16 (0.06, 0.27)
Demographics	0.37^*^ (0.33, 0.41)	0.21 (0.18, 0.24)	0.02 (0.00, 0.03)	0.18 (0.07, 0.29)	0.11 (0.05, 0.17)	-0.01^*^ (-0.03, 0.01)	0.14 (0.01, 0.28)
Demographics + employment	0.35^*^ (0.30, 0.39)	0.19 (0.16, 0.23)	0.01 (0.00, 0.02)	0.11 (0.02, 0.21)	0.09 (0.03, 0.15)	0.04 (0.03, 0.05)	0.13 (0.01, 0.26)
*By highest maternal qualification*							
Controls:							
None	0.45^*^ (0.42, 0.48)	0.23 (0.20, 0.26)	0.02^*^ (0.01, 0.02)	0.23 (0.15, 0.30)	0.12 (0.08, 0.15)	0.05^*^ (0.04, 0.07)	0.18 (0.09, 0.28)
Demographics	0.39^*^ (0.35, 0.42)	0.21 (0.18, 0.24)	0.02^*^ (0.01, 0.03)	0.17 (0.08, 0.27)	0.11 (0.06, 0.16)	0.06 (0.04, 0.07)	0.16 (0.04, 0.28)
Demographics + employment	0.36^*^ (0.32, 0.40)	0.19 (0.16, 0.22)	0.01^*^ (0.00, 0.02)	0.11 (0.03, 0.19)	0.09 (0.04, 0.14)	0.05 (0.04, 0.06)	0.13 (0.02, 0.25)
*N*	8050	15,104	10,139	2472	4785	26,153	–

95% confidence intervals in parentheses

^*^indicates significant difference from the 6-country average (*p* < .05). Demographic controls are: family structure; foreign-born parent; maternal age at birth. Employment controls are for no work, part-time or full-time maternal employment. See Table 3 for details. Estimates are average marginal predicted probabilities derived from logistic regression models. Estimates for models conditional on demographic characteristics are constructed for the reference case of: a family with two biological parents; no foreign-born parent; and a mother aged 25–29 at the child’s birth. The reference case for models conditional on maternal employment additionally specifies a part-time working mother at age 3–4. Multiple imputation is used to account for missing data on covariates

## Results

We might expect cross-country differences in the overall levels or rates of family resources (and this is indeed confirmed in the descriptive statistics by country shown in Table 3). What matters for childhood inequalities, however, is the extent to which resources differ by parental education within countries. Differences in parental education will undoubtedly have a direct effect on children but this association will be magnified to varying degrees depending on the correlation between parental education and other family resources and characteristics.

### Gaps in Income

Table 4 displays the gaps by parental education in the log of family income. In addition to displaying results for each country, Table 4 also shows the average across the six countries and indicates where a country result is significantly different from that average^6^.

Four main findings stand out in Table 4. First, the total H/L gaps in income are largest in the USA and the UK, intermediate in the Netherlands and Germany, and smallest in France and Japan. Second, adjustment for the demographic composition of the education groups explains some of the cross-national variation in the income gaps. Relative to the unconditional gaps by highest parental education, the conditional gaps decrease most in the UK, the Netherlands and Germany (by 0.29 to 0.34 log points) and least in Japan and France (by 0.08 and 0.12 log points respectively). Differences between countries become more compressed and the gaps in the UK and France no longer differ significantly from the six-country average. Nevertheless, the USA and Japan continue to stand out as the countries with significantly above and below average income gaps respectively. Third, the stratification of maternal employment by educational level differs in its importance in explaining income gaps across countries. Lower employment levels of mothers in low-educated families account for 0.12 log points of the remaining gap in the Netherlands, 0.05 to 0.07 points in the UK, France and Germany, but just 0.02 points in the US. Uniquely in Japan, mothers in high-educated families work slightly less than those in low-educated families, such that the gap becomes slightly larger when maternal employment is held constant. After adjusting for both demographic composition and maternal employment, the USA is the only country in which the H/L gap in income differs significantly from the cross-country average. Fourth, if we define parental education referring only to the mother’s education rather than that of both parents, gaps are uniformly smaller, with the average unconditional H/L gap in log income falling from 0.77 to 0.72 points. The exclusion of information about the partner’s education attenuates the unconditional gap to the greatest extent in the UK (a drop of 0.14 points) and to the least extent in Japan (a drop of 0.02 points) with a reduction of between 0.03 and 0.05 points in the other four countries. Despite this variation in the impact of changing the definition of parental education, conclusions about the pattern of country differences in income disparities are unchanged. (Appendix Table A4 provides results for H/L gaps after including additional country-specific controls such as race/ethnicity. Appendix Tables A5 and A6 provide results for H/M and M/L gaps, respectively.)

### Gaps in Center-Based Child Care

**Fig. 1:**
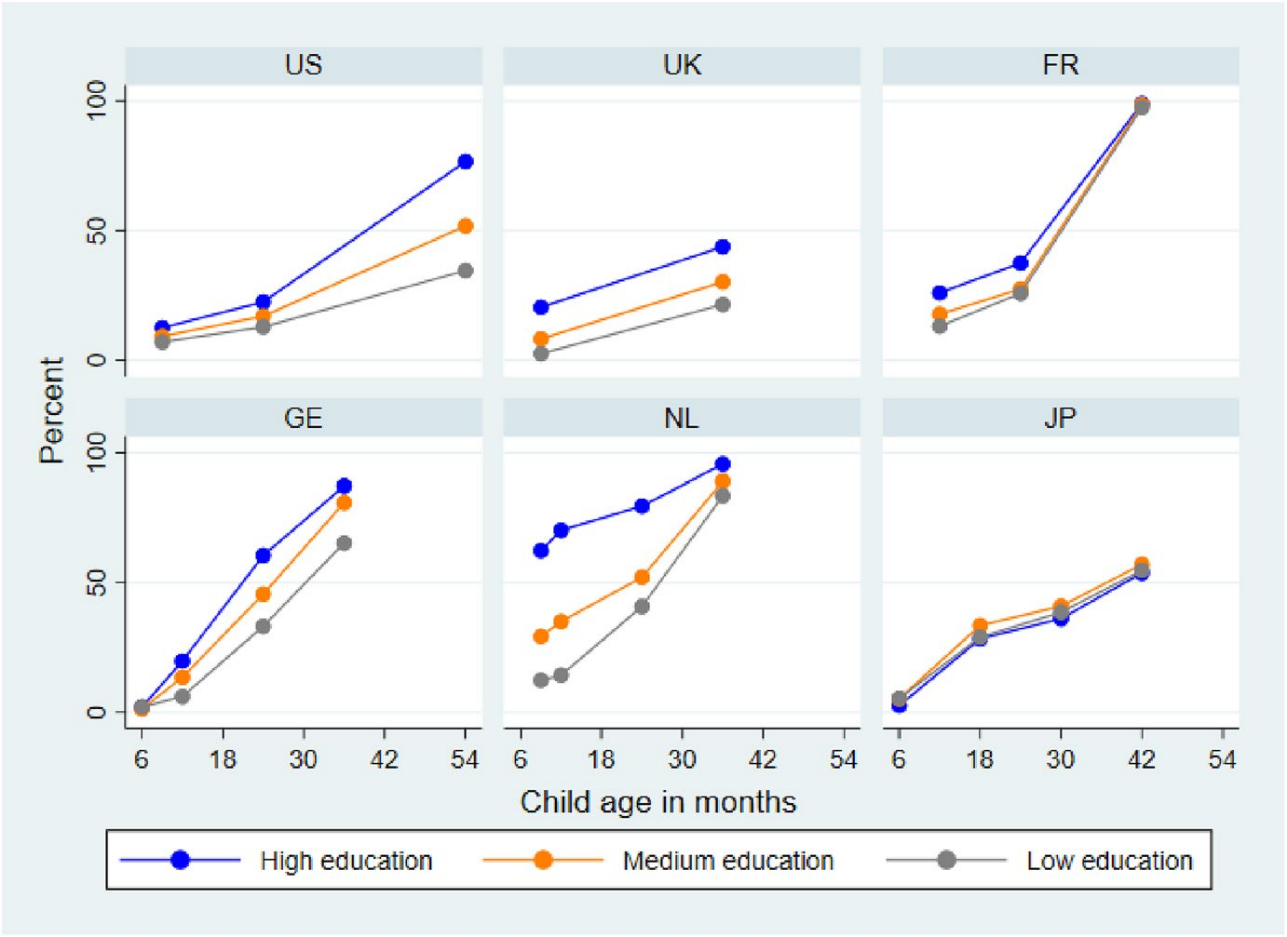
Variation by parental education in center-based care in the first 3–4 years of life

Figure 1 plots the trends by highest parental education in attendance at center-based child care from birth to age 3–4 (drawing on all available measurements up to and including the age 3–4 target wave used in this study). It is clear that experiences of center-based child care vary greatly across countries and by parental education, particularly in the age 0–2 period. Center-based care becomes virtually universal in the Netherlands by 36 months and in France by 42 months, and is experienced by the majority of children in Germany by 36 months. In contrast, sizable fractions of children in the US, the UK and Japan have not experienced center-based care by this age^7^. Prior to age 3 there is considerable variation in the age at which children in different countries and parental education groups first enroll. There are also marked differences in the degree of variation by parental education, with very large gaps by parental education in the Netherlands before 36 months and very small ones in France and Japan. The USA stands out from the other countries with a distinctive pattern of social gradients widening with child age as use of center-based child care becomes more common, reflecting its heavy reliance on parent payments for such care.

Table 5 provides the results for gaps by parental education in center-based child care at age 3–4. Four findings stand out. First, H/L gaps in center-based care by combined parental education are highest in the USA (and significantly above the six-country average), followed by the UK and Germany where gaps are moderate, then the Netherlands where gaps are small, and finally France and Japan where gaps are essentially zero (and both significantly below the country average). Second, controlling for demographic characteristics in some cases alters the size of the gaps but not the country patterning, although the gap for France becomes non-significant relative to the country average. Third, in most cases the lower employment rates of mothers in low-educated families in Western societies do surprisingly little to account for their lower utilization of center-based child care. The exception is Germany, where education-related employment differences can account for 39% of the remaining gap. Although the unconditional gap in Germany is twice the size of the gap in its neighbor the Netherlands, this differential can be almost entirely accounted for by demographic and employment-related differences. In Japan, higher rates of maternal employment in the low- relative to the high-educated group explain why center-based child care participation is actually higher in the former group. Without this offsetting influence, it is predicted that a gap of 4 percentage points in favor of the higher group would be observed, more similar to the other countries. Fourth, in contrast to the results for income, using maternal education vs highest parental education as the measure of family disadvantage results in stronger, rather than weaker, estimated disparities on average, increasing the six-country average gap from 16 to 18 percentage points. The increment in the raw maternal education gaps compared to the combined parental education gaps is small in the four European countries but notably larger by 3 percentage points in the USA and by 6 percentage points in Japan. The raw Japanese gap reverses in sign, with children in high-educated families being 1 percentage point less likely to participate in center-based care than low-educated families when the combined parent measure is used, but 5 percentage points more likely to participate when the maternal education is used. We see this as linked to the stratification of maternal employment patterns in Japan, because the difference in the gaps in third set of models, where maternal employment is controlled, is negligible. The implication is that Japanese mothers with higher-educated partners are less likely to work than their counterparts with lower-educated partners, and so less likely to rely on center-based child care. When this difference is accounted for (third row of the top panel in Table 5), a gap in favor of participation by children in high-educated families emerges, in line with the other five countries. (Appendix Table A7 provides results for H/L gaps after including additional country-specific controls. Appendix Tables A8 and A9 provide results for H/M and M/L gaps, respectively. Appendix Table A10 provides further detail about maternal employment by parental education.)

## Discussion and Conclusion

Overall, we find that the associations between parental education and key resources in early childhood are not uniform but rather vary considerably across countries. Based on the unconditional estimates much, but not all, of that variation is in line with our hypothesis that equalizing social policy regimes will moderate gaps in resources between more- and less-educated families. As expected, the two Anglo-American countries—the USA and UK – display the largest income gaps between low- and high-educated families, in line with their less generous safety nets and higher levels of income inequality and child poverty, while the three Continental European countries—France in particular—display significantly lower gaps. However, results for Japan are an anomaly—as it has both high income inequality and child poverty in national statistics but low gaps in family income between the low- and high-educated in our sample. Japan, therefore, represents a country in which the between-education-group component of income inequality appears low relative to the within-group component, at least among the population of families with young children. We think this anomaly reflects the fact that in Japan factors other than parental education (e.g., job tenure) are more consequential in driving income gaps (Kambayashi et al., 2008; Kawaguchi & Mori, 2016; OECD, 2012). It may also reflect selection into marriage (Fukuda et al., 2020).

Cross-national differences in incomes can partly be accounted for by differences in demographic composition and stratification across countries: when maternal age, family structure and migrant status are accounted for, the variation in income gaps becomes more compressed. A further minor part of the cross-national variation is linked to maternal employment: controlling for employment has the biggest impact in two of the three countries with the largest income gaps (the UK and Germany). These factors may themselves be influenced by the national policy environment in a broad sense, for example via immigration, health care and employment policies, but they are less clearly linked to the welfare safety net. What stands out clearly is that incomes in families of preschool-age children in the USA are more stratified by parental education than in the other five countries and this differential becomes starker when demographic and maternal employment explanations are accounted for.

With regard to center-based child care, there is clearly a great deal of variation in child care arrangements across the six countries, with the results lining up quite well with our hypothesis about the moderating role of social policy. We find the largest H/L gap in center-based care participation at age 3–4 in the US, consistent with its low rate of public expenditure in this area. In contrast, France with its high rate of expenditure achieves equitable use of center-based care (in the form of preschool, i.e., ecole maternelle)—with no gap by parental education. The remaining European countries display moderate gaps in line with their intermediate rates of spending, with an important explanatory role for demographic and employment-related differences in Germany only. Again, the results for Japan contrast sharply with those from Western countries. Despite an internationally-low level of ECEC spending (only just over half the OECD average), education-related gaps in center-based care are non-existent. The results suggest this is partly linked to the lower employment rates of low- and medium-educated mothers partnered with high-educated men, a group that makes up a larger fraction of the population in Japan than any of the other five countries (24.4% of all families, Appendix Table A2). Nevertheless, even when this factor is accounted for, as is the case with income, Japan provides an example of a country in which resources in the early years are relatively equally distributed by parental education. It is a question of interest for future research whether other parental socioeconomic characteristics, such as those related to occupation, are more important than education for stratification of childhood environments in Japan compared with other countries.

Our results linking high early years inequality in the USA with its comparatively limited social policies are consistent with those of Bradbury et al., (2015; for income and other resources) and Crosnoe et al., (2021; for center-based care), who also found the largest gaps by parental education in the US. However, those studies included only Anglo-American countries. Our addition of three Continental European countries and one East Asian country, with their range of social policies, is an important extension.

Due to data limitations, we are not able to trace through the implications of differences in resource disparities to disparities in developmental outcomes across countries. Our assumption is that, all else equal, smaller disparities in income and center-based childcare participation by parental education will translate into smaller disparities in children’s outcomes, conceived broadly to include cognitive, socioemotional and/or physical development. We recognize that the implications of a given resource disparity for children may depend on the absolute resource levels of different groups. Evidence suggests that the benefits to both higher income (Gershoff et al., 2007; Løken et al., 2012) and increased access to early education (Burger, 2010; Raudenbush & Eschmann, 2015) are higher for less advantaged families, although it is also possible that resources such as early education confer larger benefits to the more advantaged, as is posited in the idea that “skills beget skills” (Cunha et al., 2006), as mentioned earlier. Availability of comparative data on early childhood outcomes would enable us to test whether the consequences of gaps in income and childcare differ across countries.

An innovative aspect of our study is the use of two alternative measures of parental education. Most prior studies (including Crosnoe et al., 2021) used maternal education, on the grounds that the mother typically spends the most time with the child and is responsible for arranging child care and other inputs. In addition, datasets often lack information about the other parent. In contrast, Bradbury et al. (2015) used highest parental education, arguing that it better captured the resources and advantages available to a family in those instances where the spouse or partner had a higher level of education than the mother.

Our results using the two measures suggest that the most appropriate measure to use may depend on the outcome considered. With regard to family income, it is clear that measuring family advantage by maternal education attenuates estimates of disparities in general but also to an extent that can vary across countries, as illustrated by the disproportionately large difference in the UK estimates. With regard to child care, estimates of disparities are instead slightly larger than under the combined parental definition of education, consistent with evidence that maternal education is a particularly important determinant of child care mode and quality (Kulic et al, 2019), but again sensitivity of the estimates to the difference in definition depends on country context. The different implications of choice of household education measure across outcomes and countries, therefore, suggest that some thought is needed on the part of researchers, particularly those working in a cross-national context, about which measure will be most appropriate and why. The two measures seem to give relatively similar estimates in countries in which assortative mating and full-time maternal employment are relatively high, like France and the US, and diverge more in countries where assortative mating and full-time maternal employment are lower (patterns that we might think of as consistent with more traditional gender norms), like the UK and Japan. As noted earlier, given the categorical nature of our education data, we did not apply a third measure, average parental education; doing so is an important direction for future research.

Our study is not without limitations. First, although the data we use are extremely rich, there are some relevant variables that we could not include in the analysis because they are not measured in one or more of our countries. For example, we lacked consistent measures of parent activities with children (e.g., reading, playing games, outings) and family routines (e.g., meals, bedtime, television). Analysis of such variables would provide a fuller picture of children’s early experiences and should be a priority for future research, although we note that they are less amenable to policy intervention than the factors we focused on here. Second, in some of our countries, in particular the US, structural racism and discrimination lead to persistent differences in family resources and experiences, but the variables for these exposures could not be harmonized across countries. Studying the role of racism and discrimination in the links between parental education and resources is an important topic for future research. Third, our study represents wealthy countries only, so that we can compare similarly educated parents, in similar contexts. Relatedly, our study includes only six countries, because we are limited to those that have sufficiently detailed longitudinal data on young children, and in one of our countries (the Netherlands) the sample is drawn from one urban area and is thus not representative of the country as a whole. Future research should extend this type of analysis to a broader set of countries. Fourth, our samples represent specific cohorts and specific periods of time. For example, the Japanese, French and German cohorts were born after 2009 financial crises, whereas the remaining three cohorts were born earlier. Given that the economic impacts of the crises played out on different timescales in different countries, it is difficult to formulate predictions on precisely how differences in cohort timing may have affected our results, but this limitation should be borne in mind. Moreover, our characterization of the policy context in each country draws in indicators contemporaneous with the data collection and does not reflect more recent policy developments such as expansions of universal prekindergarten in the US.

In spite of these limitations, we provide some of the first systematic evidence about how young children’s resources vary depending on their parents’ education and the extent to which that social grading is buffered by social policies. The results are revealing. While social grading in early childhood is a fact of life in all six countries, the extent of that grading and how it manifests varies considerably. Moreover, social policy seems to play a role in moderating the influence of parental education on children’s resources. This is most evident with regard to policies such as universal child care provision which affect the care and early education children attend, and safety net policies which can lessen income inequality and improve family living conditions.

## Footnotes

1 OECD’s International Early Learning and Child Wellbeing Study aims to provide comparative data on 5-year-olds attending early education, but to date has surveyed children in only three countries (OECD, 2020).

2 There is a large comparative literature using PISA, PIRLS, and TIMSS but these pertain to school-age children and adolescents, while our focus here is on preschool age children.

3 Baccalauréat general in France.

4 There are a small number of cases of shared custody between two biological but non-co-residing parents in the French data; we group these with cases with two co-residing biological parents.

5 In France, French citizens born in other countries are counted as native-born. In Japan, the indicator relates to foreign nationality of a parent rather than place of birth.

6 This approach is analogous to testing country-level interaction terms in a fully pooled model. Pooling of datasets is not possible in this study due to data access restrictions. The unweighted cross-country average treats the individual countries’ education-related gaps as independent random variables with means and standard errors taken from the regression estimates ${\beta}_{j}$ and ${\sigma }_{j}$ respectively, $j=1,\dots ,6$ countries. The average statistics are therefore given by $\overline{\beta }=1/6{\sum }_{j=1}^{6}{\beta }_{j}$ and $\tilde{\sigma }=1/6\sqrt{{\sum }_{j=1}^{6}{\sigma }_{j}^{2}}$

7 The relatively low percentages for the UK and Japan in the Figure reflect the early timing of the age 3 survey waves, whereby many children had not made the transition to nursery (UK) or junior kindergarten (Japan) that is expected by their fourth birthday. Data from later survey waves show that 86% of the UK sample had experienced centre-based care by 48 months. Similarly, 91% of the Japanese sample had experienced centre-based care by 54 months.
